# Time-to-Onset Analysis of Drug-Induced Long QT Syndrome Based on a Spontaneous Reporting System for Adverse Drug Events

**DOI:** 10.1371/journal.pone.0164309

**Published:** 2016-10-10

**Authors:** Sayaka Sasaoka, Toshinobu Matsui, Yuuki Hane, Junko Abe, Natsumi Ueda, Yumi Motooka, Haruna Hatahira, Akiho Fukuda, Misa Naganuma, Shiori Hasegawa, Yasutomi Kinosada, Mitsuhiro Nakamura

**Affiliations:** 1 Laboratory of Drug Informatics, Gifu Pharmaceutical University, Gifu-shi, Gifu, Japan; 2 Medical database Co., LTD, Shibuya-ku, Tokyo, Japan; 3 United Graduate School of Drug Discovery and Medical Information Sciences, Gifu University, Gifu-shi, Gifu, Japan; University of Miami School of Medicine, UNITED STATES

## Abstract

Long QT syndrome (LQTS) is a disorder of the heart’s electrical activity that infrequently causes severe ventricular arrhythmias such as a type of ventricular tachycardia called torsade de pointes (TdP) and ventricular fibrillation, which can be fatal. There have been no previous reports on the time-to-onset for LQTS based on data from spontaneous reporting systems. The aim of this study was to assess the time-to-onset of LQTS according to drug treatment. We analyzed the association between 113 drugs in 37 therapeutic categories and LQTS including TdP using data obtained from the Japanese Adverse Drug Event Report database. For signal detection, we used the reporting odds ratio (ROR). Furthermore, we analyzed the time-to-onset data and assessed the hazard type using the Weibull shape parameter. The RORs (95% confidence interval) for bepridil, amiodarone, pilsicainide, nilotinib, disopyramide, arsenic trioxide, clarithromycin, cibenzoline, donepezil, famotidine, sulpiride, and nifekalant were 174.4 (148.6–204.6), 17.3 (14.7–20.4), 52.0 (43.4–62.4), 13.9 (11.5–16.7), 69.3 (55.3–86.8), 54.2 (43.2–68.0), 4.7 (3.8–5.8), 19.9 (15.9–25.0), 8.1 (6.5–10.1), 3.2 (2.5–4.1), 7.1 (5.5–9.2), and 254.8 (168.5–385.4), respectively. The medians and quartiles of time-to-onset for aprindine (oral) and bepridil were 20.0 (11.0–35.8) and 18.0 (6.0–43.0) days, respectively. The lower 95% confidence interval of the shape parameter β of bepridil was over 1 and the hazard was considered to increase over time.Our study indicated that the pattern of LQTS onset might differ among drugs. Based on these results, careful long-term observation is recommended, especially for specific drugs such as bepridil and aprindine. This information may be useful for the prevention of sudden death following LQTS and for efficient therapeutic planning.

## Introduction

Long QT syndrome (LQTS) is a disorder of the heart’s electrical activity that infrequently causes severe ventricular arrhythmias such as a type of ventricular tachycardia called torsade de pointes (TdP) and ventricular fibrillation, which can be fatal. LQTS are categorized into 2 groups: inherited (genetic) and acquired [1]. Acquired LQTS is caused by specific drugs, hypokalemia, or hypomagnesemia. Drug-induced LQTS can be caused by a variety of drugs; not only anti-arrhythmic drugs but also other non-cardiac drugs such as antipsychotics, antibiotics and anti-allergic drugs [1]. The International Council for Harmonization published “Guidance for industry E14 clinical evaluation of QT/QTc interval prolongation and proarrhythmic potential for non-antiarrhythmic drugs” in October 2005 [2]. Furthermore, the U.S. Food and Drug Administration (FDA) released a safety announcement “Azithromycin and the risk of potentially fatal heart rhythms” in May 2013. The Japanese Circulation Society joint study group published “Guidelines for Diagnosis and Management of Patients with Long QT Syndrome and Brugada Syndrome” in 2012 [1]. The Ministry of Health, Labour and Welfare in Japan released “The manual for handling disorders due to adverse drug reactions”, which is focused on ventricular tachycardia, in May 2009 [3]. Drug-induced LQTS are estimated to occur at rates of approximately 2.0–8.8% among patients prescribed anti-arrhythmic drugs and thousandth or millionth part of those prescribed non-cardiac drugs [1,4–6]. Since LQTS is a rare adverse event, epidemiologic research on the risk factors of LQTS is fraught with difficulties. Spontaneous reporting systems (SRSs), wherein clinicians report their concerns about potential drug-induced adverse events during their normal diagnostic assessments of patients, are useful for the detection of rare and severe adverse events. SRSs have been recognized as primary tools for pharmacovigilance that reflect the realities of clinical practice. In the analysis of SRS reports, data mining algorithms have been utilized to identify drug-associated adverse events by disproportionality analysis. The reporting odds ratio (ROR) has been developed for use with SRS data as a measure of the relative risk for drug-associated adverse events [7,8].

The regulatory authority in Japan, the Pharmaceuticals and Medical Devices Agency (PMDA), has released national SRS data via the Japanese Adverse Drug Event Report (JADER) database. We assessed the risk of drug-induced LQTS for prescription drugs by analyzing data obtained from the JADER database. To evaluate signals of adverse drug reactions, we used the ROR, which is commonly used in pharmacovigilance studies. It has recently been proposed that modeling the time-to-onset of adverse drug reactions could be a useful adjunct to signal detection based on relative risk [9,10]. The Weibull shape parameter (WSP) test has been suggested as a new method for the analysis of time-to-onset data without the need for a reference population [11]. The WSP can describe the varying incidence of adverse events and is used to evaluate hazard functions for detecting adverse events. There have been no previous published reports on time-to-onset analyses for LQTS using SRS data. The aims of this study were to assess the time-to-onset and ROR of LQTS according to drug treatment.

## Materials and Methods

Adverse events recorded in the JADER database from April 2004 to May 2016 were downloaded from the PMDA website (www.pmda.go.jp). The JADER database consists of 4 data tables: patient demographics information, drug information, adverse events, and primary illness. We built a relational database that integrated the 4 data tables using File Maker Pro 13 software (FileMaker, Santa Clara, CA, USA). In the “drug information” table of the JADER database, each drug was assigned a code according to its association with adverse drug reactions: “suspected drug”, “concomitant drug”, or “interacting drug.” The analysis was restricted to reports where drugs were recorded as a “suspected drug.”

The adverse events in this study relied on the definition provided by the Medical Dictionary for Regulatory Activities (MedDRA, http://www.meddra.org/) ver. 19.0. We have selected preferred terms (PTs) according to Standardized MedDRA Queries (SMQs). SMQs were built by the Maintenance and Support Services Organization. SMQs are groupings of PTs according to the level that relates to a defined medical condition, and the included terms may relate to signs, symptoms, diagnoses, syndromes, physical findings, laboratory and other physiologic test data, etc [12]. The grouping of SMQs allows for useful data retrieval and the presentation of relevant individual case safety reports. SMQs usually contain two categories of PTs corresponding to a “narrow” scope and a “broad” scope. Those two categories, “narrow” and “broad,” allow for the identification of cases that are highly likely to represent the condition of interest (a “narrow” scope) and those that would be useful when a user seeks to identify all possible cases, including some that may prove to be of little or no interest on closer inspection (a “broad” scope). The “narrow” scope yields “specificity”, while the “broad” search yields “sensitivity.” A “broad” scope includes both the “narrow” terms and the additional “broad” terms, which are often of a less-specific nature [12].

For evaluation with a “broad” scope, we utilized 20 PTs that matched the SMQ for *torsade de pointes/QT prolongation* (SMQ code: 20000001). Next, we evaluated for a “narrow” scope by utilizing a limited number of PTs that matched the same SMQ. The number of selected PTs categorized in the “narrow” scope was 6, as follows: electrocardiogram QT interval abnormal (PT code: 10063748), electrocardiogram QT prolonged (PT code: 10014387), long QT syndrome (PT code: 10024803), long QT syndrome congenital (PT code: 10057926), torsade de pointes (PT code: 10044066), and ventricular tachycardia (PT code: 10047302).

Furthermore, the possibility that drugs could shorten QT and pose safety concerns has recently become a subject of active debate [13,14]. Poluzzi *et al*. defined torsadogenicity according to the following criteria: 1) torsade de pointes, 2) QT interval abnormalities, 3) ventricular fibrillation/tachycardia, and 4) sudden cardiac death [15]. In their results, all drugs in groups 1 or 2 demonstrated a high ROR value. According to Poluzzi’s report, we selected the PTs according to groups 1 [torsade de pontes (PT code: 10044066)] and 2 [electrocardiogram QT prolonged (PT code: 10014387), long QT syndrome (PT code: 10024803), long QT syndrome congenital (PT code: 10057926), electrocardiogram QT interval abnormal (PT code: 10063748), electrocardiogram QT shortened (PT code: 10014388), electrocardiogram U-wave abnormality (PT code: 10055032), electrocardiogram U-wave biphasic (Low Level Term (LLT) code: 10055068), electrocardiogram repolarization abnormality (PT code: 10052464), and electrocardiogram R on T phenomenon (PT code: 10048883)].

Iribarren *et al*. analyzed 90 drugs [16]. They selected drugs according to the Arizona Center for Education & Research on Therapeutics website (www.azcert.org, https://crediblemeds.org) as having QT liability [13] and added carbamazepine, primidone, phenytoin, digoxin, lidocaine, and aripiprazole, which were not listed on the Arizona Center for Education & Research on Therapeutics website [14]. In this study, we selected 113 drugs in combination with the drugs analyzed by Iribarren *et al*. and according to the Japanese regulatory authority’s guidance and manual [1,3] (Table 1). Our lists include antiarrhythmic drugs and also some non-cardiac drugs.

**Table 1:** Number of reports and Reporting Odds Ratio of Long QT syndrome.

Therapeutic category	Subclassification	Drugs	Total (n)	SMQ: whole				SMQ: narrow				g1+g2 ^b^			
				Case (n)	Non-Case (n)	RR^a^(%)	ROR (95% CI)	Case (n)	Non-Case (n)	RR^a^(%)	ROR (95% CI)	Case (n)	Non-Case (n)	RR^a^(%)	ROR (95% CI)
			404450	9823	394627	2.4		2732	401718	0.7		2002	402448	0.5	
Anti-arrhythmic drug	class I	Aprindine	274	46	228	16.8	8.1 (5.9–11.2)	38	236	13.9	24.0 (17.0–33.9)	29	245	10.6	24.1 (16.4–35.5)
		Cibenzoline	749	142	607	19.0	9.5 (7.9–11.4)	87	662	11.6	19.9 (15.9–25.0)	46	703	6.1	13.4 (9.9–18.2)
		Disopyramide	363	127	236	35.0	21.9 (17.6–27.2)	113	250	31.1	69.3 (55.3–86.8)	92	271	25.3	71.5 (56.2–90.9)
		Flecainide	123	55	68	44.7	32.7 (22.9–46.6)	37	86	30.1	64.1 (43.5–94.4)	14	109	11.4	26.0 (14.9–45.4)
		Lidocaine	927	91	836	9.8	4.4 (3.5–5.5)	20	907	2.2	3.3 (2.1–5.1)	5	922	0.5	1.1 (0.5–2.6)
		Mexiletine	436	29	407	6.7	2.9 (2.0–4.2)	14	422	3.2	4.9 (2.9–8.4)	7	429	1.6	3.3 (1.6–6.9)
		Pilsicainide	648	271	377	41.8	29.7 (25.3–34.7)	162	486	25.0	52.0 (43.4–62.4)	45	603	6.9	15.3 (11.3–20.8)
		Pirmenol	46	31	15	67.4	83.3 (44.9–154.3)	27	19	58.7	211.0 (117.2–380.0)	21	25	45.7	170.6 (95.4–305.3)
		Procainamide	34	11	23	32.4	19.2 (9.4–39.5)	11	23	32.4	70.6 (34.4–145.0)	10	24	29.4	84.2 (40.2–176.3)
		Quinidine	4	0	4	0.0		0	4	0.0		0	4	0.0	
	class III	Amiodarone	1663	193	1470	11.6	5.4 (4.6–6.2)	166	1497	10.0	17.3 (14.7–20.4)	137	1526	8.2	19.3 (16.1–23.1)
		Nifekalant	97	70	27	72.2	104.9 (67.3–163.6)	61	36	62.9	254.8 (168.5–385.4)	55	42	56.7	270.7 (180.7–405.4)
		Sotalol	105	32	73	30.5	17.7 (11.7–26.8)	23	82	21.9	41.6 (26.1–66.1)	9	96	8.6	18.9 (9.5–37.5)
	class IV	Bepridil	640	365	275	57.0	55.3 (47.3–64.8)	327	313	51.1	174.4 (148.6–204.6)	293	347	45.8	198.7 (168.9–233.7)
Cardiotonic drug		Digoxin	439	62	377	14.1	6.6 (5.1–8.7)	41	398	9.3	15.4 (11.1–21.2)	25	414	5.7	12.3 (8.2–18.4)
Cardiotonic drug/antasthmatic		Epinephrine	5	0	5	0.0		0	5	0.0		0	5	0.0	
Beta blocker		Atenolol	338	40	298	11.8	5.4 (3.9–7.5)	13	325	3.8	5.9 (3.4–10.3)	11	327	3.3	6.8 (3.7–12.4)
Antipsychotics	antimanic drug	Lithium	1068	24	1044	2.2	0.9 (0.6–1.4)	9	1059	0.8	1.3 (0.6–2.4)	7	1061	0.7	1.3 (0.6–2.8)
	atypical antipsychotic	Clozapine	1062	39	1023	3.7	1.5 (1.1–2.1)	13	1049	1.2	1.8 (1.1–3.2)	13	1049	1.2	2.5 (1.4–4.3)
		Quetiapine	1893	87	1806	4.6	1.9 (1.6–2.4)	24	1869	1.3	1.9 (1.3–2.8)	17	1876	0.9	1.8 (1.1–3.0)
		Ziprasidone	1	0	1	0.0		0	1	0.0		0	1	0.0	
	banzamide	Sulpiride	1366	80	1286	5.9	2.5 (2.0–3.2)	62	1304	4.5	7.1 (5.5–9.2)	58	1308	4.2	9.1 (7.0–11.9)
	butyrophenone drug	Haloperidol	1311	97	1214	7.4	3.2 (2.6–4.0)	39	1272	3.0	4.6 (3.3–6.3)	37	1274	2.8	5.9 (4.3–8.2)
	phenothiazine antipsychotic	Chlorpromazine	1240	70	1170	5.6	2.4 (1.9–3.1)	23	1217	1.9	2.8 (1.8–4.2)	20	1220	1.6	3.3 (2.1–5.2)
		Levomepromazine	781	63	718	8.1	3.5 (2.7–4.6)	18	763	2.3	3.5 (2.2–5.6)	15	766	1.9	4.0 (2.4–6.6)
		Prochlorperazine	123	3	120	2.4	1.0 (0.3–3.2)	2	121	1.6	−^†^	1	122	0.8	−^†^
		Thioridazine	46	2	44	4.3	−^†^	1	45	2.2	−^†^	1	45	2.2	−^†^
	SSRI	Citaropram	0	0	0			0	0			0	0		
		Fluoxetine	0	0	0			0	0			0	0		
		Paroxetine	2677	95	2582	3.5	1.5 (1.2–1.8)	30	2647	1.1	1.7 (1.2–2.4)	28	2649	1.0	2.1 (1.5–3.1)
		Sertraline	976	52	924	5.3	2.3 (1.7–3.0)	17	959	1.7	2.6 (1.6–4.2)	16	960	1.6	3.4 (2.1–5.5)
		Venlafaxine	31	1	30	3.2	−^†^	0	31	0.0		0	31	0.0	
	tricyclic antidepresants	Amitriptyline	330	21	309	6.4	2.7 (1.8–4.3)	9	321	2.7	4.1 (2.1–8.0)	7	323	2.1	4.4 (2.1–9.2)
		Clomipramine	335	21	314	6.3	2.7 (1.7–4.2)	5	330	1.5	2.2 (0.9–5.4)	5	330	1.5	3.1 (1.3–7.4)
		Desipramine	0	0	0			0	0			0	0		
		Doxepin	0	0	0			0	0			0	0		
		Imipramine	264	16	248	6.1	2.6 (1.6–4.3)	3	261	1.1	1.7 (0.5–5.3)	1	263	0.4	−^†^
		Nortriptyline	39	8	31	20.5	10.4 (4.8–22.6)	3	36	7.7	12.3 (3.8–39.9)	2	37	5.1	−^†^
		Protriptyline	0	0	0			0	0			0	0		
		Trimipramine	4	2	2	50.0	−^†^	0	4	0.0		0	4	0.0	
	others	Aripiprazole	1895	84	1811	4.4	1.9 (1.5–2.3)	20	1875	1.1	1.6 (1.0–2.4)	19	1876	1.0	2.0 (1.3–3.2)
		Olanzapine	1749	105	1644	6.0	2.6 (2.1–3.1)	33	1716	1.9	2.9 (2.0–4.0)	26	1723	1.5	3.1 (2.1–4.5)
		Pimozide	44	5	39	11.4	5.2 (2.0–13.1)	5	39	11.4	18.9 (7.4–47.9)	4	40	9.1	20.1 (7.2–56.3)
		Risperidone	2604	125	2479	4.8	2.0 (1.7–2.4)	33	2571	1.3	1.9 (1.3–2.7)	31	2573	1.2	2.4 (1.7–3.5)
Antibiotics		Azithromycin	1235	60	1175	4.9	2.1 (1.6–2.7)	29	1206	2.3	3.6 (2.5–5.2)	25	1210	2.0	4.2 (2.8–6.2)
		Ciprofloxacin	737	14	723	1.9	0.8 (0.5–1.3)	9	728	1.2	1.8 (0.9–3.5)	7	730	0.9	1.9 (0.9–4.1)
		Clarithromycin	3318	145	3173	4.4	1.8 (1.6–2.2)	100	3218	3.0	4.7 (3.8–5.8)	89	3229	2.7	5.8 (4.6–7.1)
		Co-trimoxazole	0	0	0			0	0			0	0		
		Erythromycin	175	8	167	4.6	1.9 (0.9–3.9)	7	168	4.0	6.1 (2.9–13.1)	3	172	1.7	3.5 (1.1–11.0)
		Fluconazole	344	18	326	5.2	2.2 (1.4–3.6)	17	327	4.9	7.7 (4.7–12.5)	14	330	4.1	8.6 (5.0–14.7)
		Garenoxacin	1618	75	1543	4.6	2.0 (1.6–2.5)	31	1587	1.9	2.9 (2.0–4.1)	27	1591	1.7	3.4 (2.3–5.1)
		Gatifloxacin	266	4	262	1.5	0.6 (0.2–1.6)	1	265	0.4	−^†^	1	265	0.4	−^†^
		Itraconazole	1178	20	1158	1.7	0.7 (0.4–1.1)	12	1166	1.0	1.5 (0.9–2.7)	10	1168	0.8	1.7 (0.9–3.2)
		Ketoconazole	12	0	12	0.0		0	12	0.0		0	12	0.0	
		Levofloxacin	3293	52	3241	1.6	0.6 (0.5–0.8)	25	3268	0.8	1.1 (0.8–1.7)	22	3271	0.7	1.4 (0.9–2.1)
		Moxifloxacin	607	72	535	11.9	5.4 (4.2–7.0)	30	577	4.9	7.7 (5.3–11.2)	28	579	4.6	9.8 (6.7–14.4)
		Ofloxacin	104	3	101	2.9	1.2 (0.4–3.8)	0	104	0.0		0	104	0.0	
		Pentamidine	245	15	230	6.1	2.6 (1.6–4.4)	12	233	4.9	7.6 (4.3–13.6)	9	236	3.7	7.7 (3.9–15.0)
		Voriconazole	925	31	894	3.4	1.4 (1.0–2.0)	22	903	2.4	3.6 (2.4–5.5)	18	907	1.9	4.0 (2.5–6.4)
Antineoplastic agent		Arsenic trioxide	397	106	291	26.7	14.8 (11.8–18.5)	104	293	26.2	54.2 (43.2–68.0)	99	298	24.9	70.2 (55.7–88.5)
		Crizotinib	793	33	760	4.2	1.7 (1.2–2.5)	27	766	3.4	5.2 (3.6–7.7)	27	766	3.4	7.2 (4.9–10.5)
		Dasatinib	917	24	893	2.6	1.1 (0.7–1.6)	19	898	2.1	3.1 (2.0–4.9)	19	898	2.1	4.3 (2.7–6.8)
		Nilotinib	1520	138	1382	9.1	4.1 (3.4–4.8)	126	1394	8.3	13.9 (11.5–16.7)	126	1394	8.3	19.3 (16.0–23.3)
		Octreotide	402	6	396	1.5	0.6 (0.3–1.4)	0	402	0.0		0	402	0.0	
		Pazopanib	729	15	714	2.1	0.8 (0.5–1.4)	6	723	0.8	1.2 (0.5–2.7)	6	723	0.8	1.7 (0.7–3.7)
		Tamoxifen	655	7	648	1.1	0.4 (0.2–0.9)	7	648	1.1	1.6 (0.8–3.4)	7	648	1.1	2.2 (1.0–4.6)
Anti-ulcer drug	H2 recepter antagonist	Cimetidine	189	3	186	1.6	0.6 (0.2–2.0)	2	187	1.1	−^†^	2	187	1.1	−^†^
		Famotidine	3168	87	3081	2.7	1.1 (0.9–1.4)	67	3101	2.1	3.2 (2.5–4.1)	63	3105	2.0	4.2 (3.2–5.4)
Anticonvulsant	Antiepileptic drug	Carbamazepine	4917	95	4822	1.9	0.8 (0.6–1.0)	9	4908	0.2	0.3 (0.1–0.5)	8	4909	0.2	0.3 (0.2–0.7)
		Primidone	25	2	23	8.0	−^†^	0	25	0.0		0	25	0.0	
	others	Lamotrigine	2794	43	2751	1.5	0.6 (0.5–0.8)	6	2788	0.2	0.3 (0.1–0.7)	6	2788	0.2	0.4 (0.2–1.0)
		Levetiracetam	957	18	939	1.9	0.8 (0.5–1.2)	2	955	0.2	−^†^	2	955	0.2	−^†^
		Phenytoin	1487	33	1454	2.2	0.9 (0.6–1.3)	9	1478	0.6	0.9 (0.5–1.7)	7	1480	0.5	1.0 (0.5–2.0)
Alzheimer's type dementia treatment drug		Donepezil	1631	202	1429	12.4	5.8 (5.0–6.7)	83	1548	5.1	8.1 (6.5–10.1)	77	1554	4.7	10.3 (8.2–13.0)
		Galantamine	481	55	426	11.4	5.2 (3.9–6.9)	6	475	1.2	1.9 (0.8–4.2)	6	475	1.2	2.5 (1.1–5.7)
Overactive bladder treatment drug	antispasmodic	Solifenacin	663	25	638	3.8	1.6 (1.1–2.4)	16	647	2.4	3.7 (2.2–6.0)	15	648	2.3	4.7 (2.8–7.8)
Antasthmatic	LABA	Salmeterol	596	11	585	1.8	0.8 (0.4–1.4)	1	595	0.2	−^†^	1	595	0.2	−^†^
	SABA	Metaproterenol	0	0	0			0	0			0	0		
		Albuterol	0	0	0			0	0			0	0		
	others	Terbutaline	0	0	0			0	0			0	0		
		Levalbuterol	0	0	0			0	0			0	0		
Antiemetic		Dolasetron	0	0	0			0	0			0	0		
		Ondansetron	21	0	21	0.0		0	21	0.0		0	21	0.0	
Antihistamine drug		Diphenhydramine	97	2	95	2.1	−^†^	0	97	0.0		0	97	0.0	
		Fexofenadine	531	25	506	4.7	2.0 (1.3–3.0)	6	525	1.1	1.7 (0.8–3.8)	6	525	1.1	2.3 (1.0–5.2)
		Hydroxyzine	350	34	316	9.7	4.3 (3.0–6.2)	11	339	3.1	4.8 (2.6–8.7)	9	341	2.6	5.3 (2.7–10.3)
Antiobesity		Fenfluramine	0	0	0			0	0			0	0		
		Phentermine	0	0	0			0	0			0	0		
		Sibutramine	0	0	0			0	0			0	0		
Cold medicine		Ephedrine	123	22	101	17.9	8.8 (5.5–13.9)	7	116	5.7	8.9 (4.1–19.1)	4	119	3.3	6.8 (2.5–18.3)
		Phenylpropanolamine	5	0	5	0.0		0	5	0.0		0	5	0.0	
		Pseudoephedrine	60	3	57	5.0	2.1 (0.7–6.8)	0	60	0.0		0	60	0.0	
Antiparkinson		Amantadine	844	25	819	3.0	1.2 (0.8–1.8)	6	838	0.7	1.1 (0.5–2.4)	6	838	0.7	1.4 (0.6–3.2)
Stimulant drug		Amphetamine	0	0	0			0	0			0	0		
Antiretroviral / protease inhibitor		Atazanavir	311	2	309	0.6	−^†^	0	311	0.0		0	311	0.0	
ADHD treatment drug		Atomoxetine	111	6	105	5.4	2.3 (1.0–5.2)	1	110	0.9	−^†^	1	110	0.9	−^†^
Lipid-lowering agent		Atorvastatin	2424	33	2391	1.4	0.6 (0.4–0.8)	7	2417	0.3	0.4 (0.2–0.9)	7	2417	0.3	0.6 (0.3–1.2)
Sedative		Chloral hydrate	29	0	29	0.0		0	29	0.0		0	29	0.0	
Antimalarial drug		Chloroquine	0	0	0			0	0			0	0		
Gastrointestinal prokinetic agent		Cisapride	1	0	1	0.0		0	1	0.0		0	1	0.0	
Diuretic		Indapamide	201	7	194	3.5	1.4 (0.7–3.1)	2	199	1.0	−^†^	2	199	1.0	−^†^
Antihypertensive drug		Isradipine	0	0	0			0	0			0	0		
Cancer pain drug		Methadone	29	5	24	17.2	8.4 (3.2–22.0)	5	24	17.2	30.7 (11.7–80.5)	5	24	17.2	42.0 (16.0–110.1)
Narcolepsy treatment drugs/ADHD treatment drug		Methylphenidate	376	13	363	3.5	1.4 (0.8–2.5)	4	372	1.1	1.6 (0.6–4.2)	2	374	0.5	−^†^
Antihypotensive drug		Midodorine	32	1	31	3.1	−^†^	1	31	3.1	−^†^	1	31	3.1	−^†^
Antihypertensive drug		Nicardipine	293	18	275	6.1	2.6 (1.6–4.2)	0	293	0.0		0	293	0.0	
Adrenergic agents		Phenylephrine	90	8	82	8.9	3.9 (1.9–8.1)	2	88	2.2	−^†^	1	89	1.1	−^†^
Antihyperlipidemic drug		Probucol	63	22	41	34.9	21.6 (12.9–36.3)	18	45	28.6	59.2 (34.2–102.4)	18	45	28.6	81.1 (46.9–140.4)
Immunosuppressant		Tacrolimus	8052	30	8022	0.4	0.1 (0.1–0.2)	4	8048	0.0	0.1 (0.0–0.2)	1	8051	0.0	−^†^
Antiallergic drug		Terfenadine	0	0	0			0	0			0	0		
α-2 adrenergic agonist / muscle relaxant		Tizanidine	271	13	258	4.8	2.0 (1.2–3.5)	6	265	2.2	3.3 (1.5–7.5)	4	267	1.5	3.0 (1.1–8.1)
Overactive bladder treatment drug		Tolterodine	106	2	104	1.9	−^†^	0	106	0.0		0	106	0.0	
Phosphodiesterase inhibitor		Vardenafil	27	3	24	11.1	5.0 (1.5–16.7)	0	27	0.0		0	27	0.0	

^a^ RR: Reporting Ratio,

^b^ PTs according to group1 and 2 defined by Poluzzi *et al*.,

^†^ Number of cases ≤2.

Among these selected drugs, anti-arrhythmic drugs were categorized using the Vaughan–Williams classification system.

For signal detection, we calculated the ROR as the ratio of the odds of reporting an adverse event (LQTS), *versus* all other events for a given drug, compared to the reporting odds for all other drugs in the JADER database. RORs were expressed as point estimates with a 95% confidence interval (CI). Safety signals were considered significant when the lower limit of the 95% CI of the estimated ROR was greater than 1 [7].

The median, quartiles, and WSP were used for evaluation of the time-to-onset data [9,10]. Time-to-onset data was calculated from the start date of administration to the occurrence of the specific adverse events. It is necessary to take right truncation into account when estimating the time-to-onset of adverse events from SRS data. We chose an analysis period of 65 days after the start of administration to focus attention on the onset of adverse events within 2 months after the drug administration. The rate of occurrence of adverse events after prescription is thought to depend on the causal mechanism and will often vary over time; in contrast, adverse events not associated with the drug will occur at a constant background rate. The WSP test is used for statistical analysis of time-to-onset data and can describe the non-constant ratio of incidence of adverse events [10,11,17]. The shape parameter β of the Weibull distribution has been used to indicate the level of hazard over time without a reference population. Accordingly, in this study, when β was equal to 1, the hazard was considered to be constant over time. When β was lower than 1, the hazard was considered to decrease over time. In contrast, when β was greater than 1, the hazard was considered to increase over time [9–11,17].

All data analyses were performed using JMP 11.0 (SAS Institute, Cary, NC, USA).

## Results

The JADER database contained 404,450 reports from April 2004 to May 2016. The number of case reports extracted by searching using the SMQ for *torsade de pointes/QT prolongation* (SMQ code: 20000001) with PTs matching a “narrow” scope, a “broad” scope, and the PTs selected by Iribarren *et al*. were 9,823 (2.4%), 2,732 (0.7%), and 2,002 (0.5%), respectively. The number of case reports, reporting ratio, and ROR including 95% CI for each drug are summarized in Table 1.

In the “narrow” scope, the RORs (95% CI) for male and female subjects were 0.79 (0.74–0.86) and 1.28 (1.19–1.38), respectively. The numbers of case reports of bepridil, amiodarone, pilsicainide, nilotinib, disopyramide, arsenic trioxide, clarithromycin, cibenzoline, donepezil, famotidine, sulpiride, and nifekalant were 327 (51.1%), 166 (10.0%), 162 (25.0%), 126 (8.3%), 113 (31.1%), 104 (26.2%), 100 (3.0%), 87 (11.6%), 83 (5.1%), 67 (2.1%), 62 (4.5%), and 61 (62.9%), respectively. The RORs (95% CI) for these drugs were 174.4 (148.6–204.6), 17.3 (14.7–20.4), 52.0 (43.4–62.4), 13.9 (11.5–16.7), 69.3 (55.3–86.8), 54.2 (43.2–68.0), 4.7 (3.8–5.8), 19.9 (15.9–25.0), 8.1 (6.5–10.1), 3.2 (2.5–4.1), 7.1 (5.5–9.2), and 254.8 (168.5–385.4), respectively. The 95% CI lower limit of these drugs significantly exceeded 1, and so the signal for association with LQTS was considered to be significant for these drugs.

The time-to-onset data and WSP analyses are summarized in Table 2.

**Table 2:** The medians and weibull parameter of each drugs.

Therapeutic category	Drugs		Case reports^a^	Median (day) (25%-75%)	Scale parameter	Shape parameter
					α (95% CI)	β (95% CI)
Anti-arrhythmic drug	Total (oral)		258	11.0 (3.0–31.3)	21.3 (18.5–24.3)	1.0 (0.9–1.1)
		disopyramide (oral)	17	3.0 (1.0–22.0)	9.3 (4.0–20.5)	0.7 (0.4–0.9)
		flecainide (oral)	14	9.0 (4.8–12.5)	13.0 (8.0–20.7)	1.3 (0.8–1.8)
		pilsicainide (oral)	39	4.0 (3.0–24.0)	12.9 (8.4–19.4)	0.8 (0.7–1.1)
		cibenzoline (oral)	21	5.0 (2.0–10.5)	10.1 (5.8–17.0)	0.9 (0.6–1.2)
		aprindine (oral)	16	20.0 (11.0–35.8)	20.5 (12.8–31.9)	1.3 (0.8–1.8)
		amiodarone (oral)	23	3.0 (0.0–12.0)	13.7 (6.5–27.6)	0.8 (0.5–1.1)
		bepridil (oral)	128	18.0 (6.0–43.0)	30.7 (26.6–35.3)	1.4 (1.2–1.6)
	Total (i.v.)		80	0.0 (0.0–1.0)	2.7 (1.8–3.8)	1.0 (0.8–1.2)
		pilsicainide (i.v.)	14	0.0 (0.0–0.0)	–^†^	–^†^
		amiodarone (i.v.)	20	0.0 (0.0–1.8)	3.3 (1.4–7.3)	0.9 (0.6–1.4)
		nifekalant (i.v.)	37	1.0 (0.0–1.0)	2.6 (1.6–4.2)	1.0 (0.7–1.3)
		others (i.v.)	9	0.0 (0.0–0.5)	1.7 (0.8–3.7)	3.5 (0.8–9.3)
		disopyramide (i.v.) flecainide (i.v.) cibenzoline (i.v.) aprindine (i.v.)	3 3 2 1	0.0 (0.0–0.0) 0.0 (0.0–0.0) 0.5 (0.0–1.0) 2.0 (2.0–2.0)	–^†^ –^†^ –^†^ –^†^	–^†^ –^†^ –^†^ –^†^
Digitalis preparation		digoxin (oral)	5	9.0 (3.0–24.5)	17.6 (4.3–70.2)	1.2 (0.4–2.6)
		digoxin (i.v.)	1	1.0 (1.0–1.0)	–^†^	–^†^
Antipsychotics	Total		47	10.0 (3.0–19.0)	16.9 (12.3–22.8)	1.1 (0.8–1.3)
		haloperidol (oral or i.m.)	5	16.0 (8.0–49.0)	36.9 (18.9–70.0)	2.1 (0.8–4.4)
		haloperidol (i.v.)	8	11.0 (3.0–19.0)	10.4 (3.6–28.2)	0.9 (0.4–1.6)
		olanzapine (oral or i.m.)	7	10.0 (2.0–29.0)	16.0 (6.7–36.2)	1.2 (0.6–2.1)
		paroxetine (oral)	6	3.5 (0.8–47.8)	17.3 (3.2–84.0)	0.7 (0.3–1.3)
		sulpiride (oral)	11	10.0 (8.0–27.0)	18.7 (9.7–34.6)	1.1 (0.7–1.8)
		risperidone (oral)	10	11.5 (1.0–16.0)	11.2 (6.2–19.5)	1.4 (0.7–2.4)
Antibiotics		clarithromycin (oral)	37	7.0 (3.0–21.0)	13.5 (9.7–18.6)	1.1 (0.9–1.5)
		garenoxacin (oral)	24	3.0 (1.0–4.0)	3.5 (2.7–4.3)	2.2 (1.5–3.0)
		moxifloxacin (oral)	27	0.0 (0.0–6.0)	6.8 (3.8–11.7)	1.1 (0.7–1.6)
Anti-ulcer drug		famotidine (oral)	11	10.0 (2.0–20.0)	16.7 (7.8–34.1)	1.0 (0.6–1.5)
		famotidine (i.v.)	6	10.5 (2.3–30.3)	20.7 (7.7–52.8)	1.2 (0.5–2.2)
Anticancer drug		arsenic trioxide (i.v.)	50	11.0 (4.0–18.8)	14.7 (11.3–18.9)	1.2 (0.9–1.5)
		nilotinib (oral)	27	8.0 (2.0–18.0)	15.4 (10.9–21.3)	1.4 (1.0–1.9)
Curative drug for Alzheimer’s		donepezil (oral or i.n.)	13	14.0 (1.5–30.0)	17.0 (8.3–33.1)	1.0 (0.6–1.5)

^a^ Number of reports used for analysis.

^†^ Number of cases with different time-to-onset durations ≤3.

**Fig 1:**
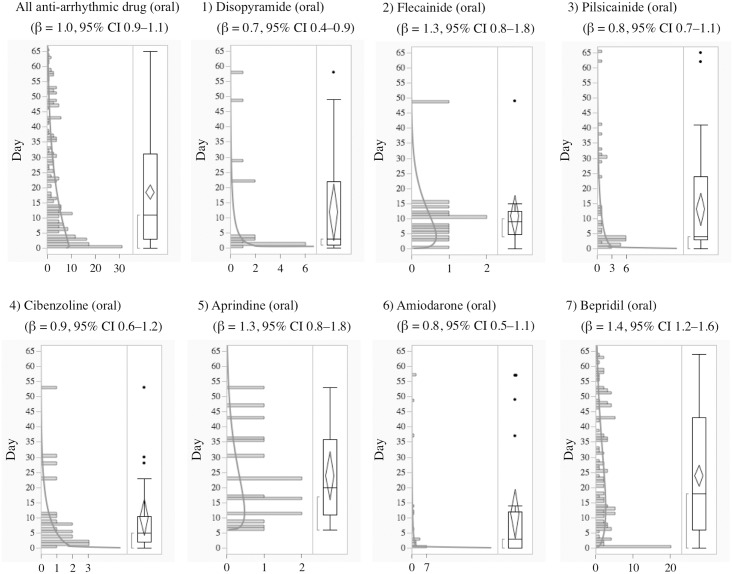
Histogram and Weibull Shape Parameter of Long QT Syndrome for 1) Disopyramide (oral), 2) Flecainide (oral), 3) Pilsicainide (oral), 4) Cibenzoline (oral), 5) Aprindine (oral), 6) Amiodarone (oral), 7) Bepridil (oral).

**Fig 2:**
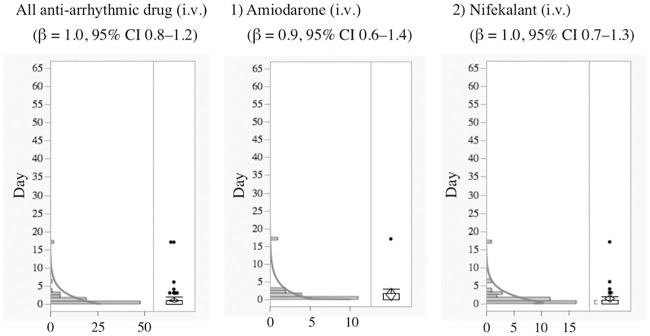
Histogram and Weibull Shape Parameter of Long QT Syndrome for 1) Amiodarone (i.v.), 2) Nifekalant (i.v.).

**Fig 3:**
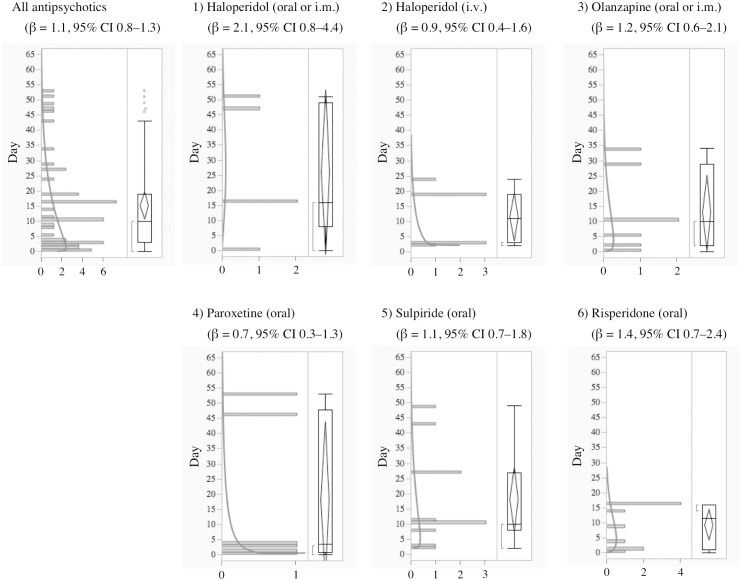
Histogram and Weibull Shape Parameter of Long QT Syndrome for 1) Haloperidol (oral or i.m.), 2) Haloperidol (i.v.), 3) Olanzapine (oral or i.m.), 4) Paroxetine (oral), 5) Sulpiride (oral), 6) Risperidone (oral).

**Fig 4:**
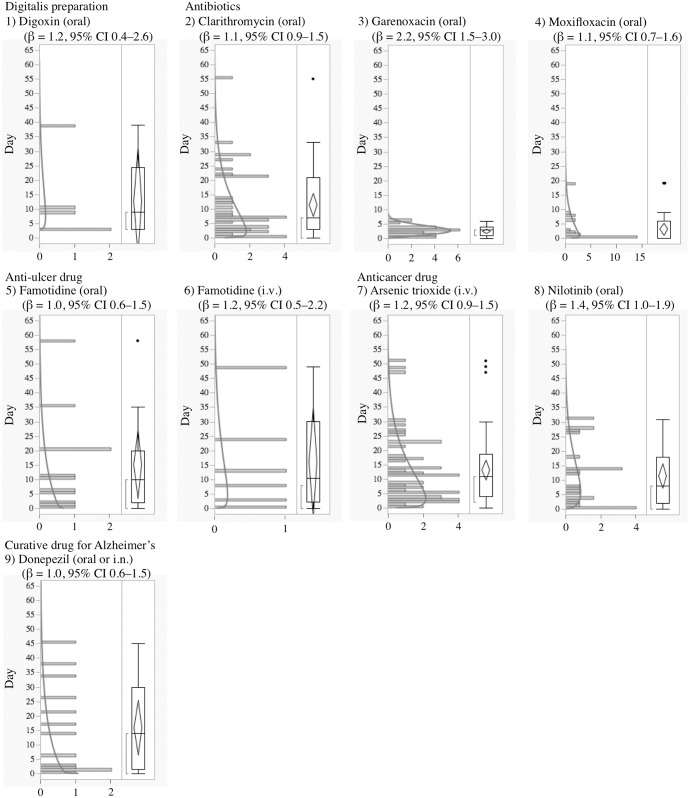
Histogram and Weibull Shape Parameter of Long QT Syndrome for 1) Digoxin (oral), 2) Clarithromycin (oral), 3) Garenoxacin (oral), 4) Moxifloxacin (oral), 5) Famotidine (oral), 6) Famotidine (i.v.), 7) Arsenic trioxide (i.v.), 8) Nilotinib (oral), 9) Donepezil (oral or i.n.).

The median and quartiles of anti-arrhythmic drugs (oral) and anti-arrhythmic drugs (intravenous) were 11.0 (3.0–31.3) and 0.0 (0.0–1.0) days, respectively. The median and the quartiles of aprindine (oral) and bepridil were 20.0 (11.0–35.8) and 18.0 (6.0–43.0) days, respectively. The time-to-onset profiles are demonstrated in Figs 1–4.

The WSP β and 95% CI lower limit of bepridil exceeded 1, indicating a significant association of bepridil with LQTS.

## Discussion

The high frequency and unpredictability of drug-induced LQTS are important issues for clinicians. The most common cause of drug-induced LQTS is inhibition of the cardiac hERG potassium channel [18,19]. It has been known that several anti-arrhythmic drugs also have a risk of causing a pro-arrhythmic effect [20], which is a problem that has been reported in clinical practice [3]. Our analysis showed that all of the anti-arrhythmic drugs we evaluated had high and significant RORs in the “narrow” scope (Table 1). In particular, anti-arrhythmic drugs belonging to Vaughan*–*Williams classification category 3, which have a K channel blocking action and are used when other anti-arrhythmic drugs are not effective, had especially high RORs. The occurrence rates of ventricular tachycardia (including TdP) described in the packaging inserts of pilsicainide, aprindine, and bepridil are “0.31%”, “0.20%”, and “0.20%”, respectively. For nifekalant, the occurrence rate of ventricular tachycardia (including TdP) is described as “more than 5%”. In comparison with other anti-arrhythmic drugs, the LQTS risk of nifekalant might be high. Upon further consideration of the mechanism (cardiac hERG potassium channel inhibitory effect) underlying QT prolongation, the high ROR of nifekalant that we detected was assumed to be a plausible result.

Drug-induced LQTS has led to withdrawals or restrictions on the use of a number of marketed drugs over the last decade, especially non-cardiovascular drugs [21,22]. In our analysis, significant RORs were detected for 34 non-cardiovascular drugs, including pimozide, arsenic trioxide, nilotinib, donepezil, ephedrine, methadone, probucol, etc. (Table 1). These 34 drugs have been reported to potentially cause QT prolongation by several studies [16,23–25]. The results obtained by analysis of data from the JADER database in this study were reasonable in the context of those reported in the literature. For bepridil, amiodarone, nilotinib, arsenic trioxide, and nifekalant, warnings about the risk of LQTS or ventricular tachycardia including TdP are described in the warning boxes of their package inserts in Japanese.

The risk of LQTS is known to be increased by many factors, including: female gender, cardiac hypertrophy, chronic heart failure, cardiomyopathies, electrolyte imbalance (hypokalemia and hypomagnesemia), polypharmacy, race, etc. [21,22]. Gender was reported as both a demographic and systematic risk factor of LQTS [3,21,22]. A potential interaction of quinidine with gender was reported [26,27]. The RORs in female subjects were higher than those in male subjects were. A reduced cardiac repolarization reserve closely related to sex steroids has been proposed to explain the higher propensity of women to develop drug-induced LQTS [22]. Polypharmacy giving rise to drug-drug interactions should also be considered as a risk factor for drug-induced LQTS [22,28–30]. For the majority of drugs except for class IA drugs, LQTS risk increases with the dose prescribed [19]. The results of a retrospective analysis of the FDA Adverse Event Reporting System database (FAERS) by Shaffer *et al*., where concomitant risk factors for QT prolongation and TdP occurring in association with the administration of macrolide antimicrobials were examined, showed that the co-administration of drugs prolonging the QT interval accounts for 50% of registered TdP reports [31]. Pharmacokinetic interactions with drugs, such as thioridazine, erythromycin, and terfenadine, that are known to inhibit cytochrome P450 isozyme CYP3A4 can enhance the LQTS risk of these agents by decreasing their clearance [3,19]. Elderly female patients with polypharmacy including drugs with the potential to inhibit drug-elimination mechanisms are at a particularly increased risk. Certain drugs (e.g., diuretics that cause hypokalemia) inhibit drugs elimination [21]. The acquired factor of age might influence the ROR. More detailed analysis focusing on these factors is a subject for future investigation.

Curtis and colleagues have evaluated the concomitant use of QT prolonging drugs in a cohort of 1.1 million patients. The concomitant use of 2 QT prolonging drugs or a QT prolonging drug and a drug that inhibits its clearance was identified in 9.4% of patients, and the concomitant use of ≥ 3 drugs was observed in 0.7% of patients [32]. We surveyed the co-administration frequencies among 113 drugs in this study. From the evaluation utilizing the SMQ with a “narrow” scope, we obtained frequencies as follows for monotherapy with anti-arrhythmic drugs [aprindine (31.6% [12/38]), disopyramide (73.5% [83/113]), cibenzoline (82.8% [72/87]), nifekalant (88.5% [54/61]), pilsicainide (87.7% [142/162]), and bepridil (75.5% [247/327])] and antipsychotics [haloperidol (25.6% [10/39]), chlorpromazine (17.4% [4/23]), and sulpiride (48.4% [30/62])]. From these data, it seems that the frequency of co-administration of anti-arrhythmic drugs with other drugs that could potentially induce LQTS was high. On the contrary, it seems that the frequency of co-administration of antipsychotics with other drugs was low. Despite being a potent antiarrhythmic and causing prolongation of the QT interval, amiodarone appears to be associated with a low frequency of pro-arrhythmic events [33]. In our study, the number of reports and the ROR of amiodarone were relatively high. We do not have a conclusive explanation for these data. We could consider that the co-administration of amiodarone and other drug(s) partially explains the high values. We did not examine the potential drug-by-drug bias because there were too few cases for the robust further analysis of time-to-onset.

Several studies with the FAERS database, which is an SRS database, have demonstrated significant associations between LQTS and certain suspected drugs [15,34,35]. The FAERS database is a computerized SRS information system to which healthcare professionals and consumers send adverse event reports voluntarily through the MedWatch program. Poluzzi *et al*. evaluated antipsychotics and torsadogenic risk using the FAERS database and identified 3 torsadogenic signals for amisulpride, cyamemazine, and olanzapine, which were neither mentioned by the 2011 AZCERT classifications nor arose from previous literature data [15]. Validation and risk quantification through different sources such as healthcare databases are generally recommended in the evaluation of SRS data [15]. The torsadogenic risks of oral antihistamines and antipsychotics were evaluated by combining the FAERS database with drug utilization data across Europe [34,35]. Based on the JADER database, Ikeno *et al*. reported a relationship between antipsychotics and the cardiac adverse events of QT prolonged and TdP [36]. Significant RORs for associations of the antipsychotic drugs haloperidol and sulpiride with LQTS were observed in our study.

To the best of our knowledge, no time-to-onset analyses for drug-induced LQTS have been systematically addressed using SRSs. Although the analysis strategy carries the inherent limitations of SRS data, the aim of our time-to-onset analysis was to obtain new information and compare the relative risks and onset profiles of LQTS for prescription drugs.

Generally, anti-arrhythmic drugs that inhibit the hERG potassium channel cause electrophysiological changes immediately following administration, and LQTS and TdP occur a few days later except for specific drugs, such as probucol and bepridil [3]. In the time-to-onset analysis, the medians of times-to-onset for anti-arrhythmic drugs (intravenous) were within 1 day of each other. Special attention should be paid to the possibility of LQTS onset with these drugs and careful observation is recommended from soon after administration until 1–2 days, especially for the intravenous route of administration. The profiles of aprindine and bepridil were different from those of other anti-arrhythmic drugs (Table 2, Figs 1 and 2). According to the package inserts and interview forms, the half-lives of drug elimination for aprindine (50 h) and bepridil (80 h) are longer than those for other anti-arrhythmic drugs (2–13.4 h). It has been reported that the LQTS risk of bepridil often increases depending on time [3]. The medians times-to-onset for aprindine (oral) and bepridil (oral) were 20 and 18 days, respectively (Table 2). The WSP β of bepridil was 1.4 (1.2–1.6) and so the hazard was considered to increase over time (Table 2). The medians times-to-onset for antipsychotics and arsenic trioxide were 10.0 and 11.0 days, respectively (Table 2). These results corresponded with those of previous reports and confirmed the necessity of long-term observation after the administration of these drugs.

In acquired LQTS, latent genetic backgrounds may be related to the prolongation of the QT interval [37,38]. Five to nineteen percent of patients with drug-induced torsade de pointes carry mutations in genes involved in congenital long QT syndrome [39,40]. For congenital LQTS, as many as 13 distinct genetic mutations have been identified [41]. Several potassium voltage-gated channel subfamily H member 2 (*KCNH2*) mutations were identified in persons with drug-induced arrhythmias in a database of SRS for adverse drug reactions [42]. Recently, pharmacokinetic and pharmacodynamic influencing polymorphisms related to drug-induced QT interval prolongation have been detected by genome-wide association studies [38,43]. The frequencies of polymorphisms in genes known to be associated with congenital long QT syndrome had varying distributions among ethnic groups [38]. Caucasians seem to be more sensitive to QT prolongation than other ethnicities [38]. Our analysis did not consider this genetic factor because the JADER database lacks detailed clinical information [44]. Thus, typical of results obtained from analysis using SRS databases, our results require further epidemiological studies for confirmation.

In our results, the RORs of several drugs such as nifekalant, bepridil, pirmenol, etc. had huge values (> 100) in the case of analysis with the PTs group of the “narrow” scope or with the PTs group of “g1 + g2” compared to the results with the “broad” scope (Table 1). The calculated RORs might vary significantly depending on the selection of PTs.

Electrocardiography (ECG) is an important criterion for the diagnosis of torsade de pointes/QT prolongation. Iribarren *et al*. have assessed the drug-induced alteration of QT interval in a self-controlled crossover study using a large ECG database [16]. Since detailed patient information such as ECG is not intrinsically included in the JADER database, we did not evaluate ECG results in this study.

In this study, we selected PTs for the identification of torsade de pointes/QT prolongation, which is coded according to the terminology prescribed by the MedDRA. According to the Introductory Guide MedDRA Version 19.0, each PT is a distinct descriptor (a single medical concept) for a symptom, sign, disease, diagnosis, therapeutic indication, investigation, surgical, or medical procedure, or a medical, social, or family history characteristic [45]. PTs should be unambiguous and as specific and self-descriptive as possible in the context of international requirements. Therefore, eponymous terms are only used when they are recognized internationally. The granularity/specificity of the PT level is such that clinical pathologic or etiologic qualifiers of the descriptors are represented at the PT level (http://www.meddra.org/sites/default/files/guidance/file/intguide_19_0_english.pdf) [45]. The JADER database is derived from spontaneous volunteer reporting. The contributors only report adverse events according to ICH E2B, the international safety reporting guidelines, and rely on the definitions provided by MedDRA. It was difficult to confirm the criteria used to define torsade de pointes/QT prolongation events by volunteers at the time of reporting. However, the reports in the JADER database have been reported by healthcare “professionals.” Furthermore, our analysis was restricted to reports where drugs were recorded as a “suspected drug”. We consider that these data suggest the association of certain drugs with torsade de pointes/QT prolongation, but further validation of these associations is needed.

The SRS is subject to various biases, including the exclusion of healthy individuals, the lack of a denominator, and confounding factors [8]. The ROR is defined as the ratio of reported cases of a defined adverse event of interest versus all other adverse events for the drug of interest, compared to the reporting odds for all other drugs present in the database. In basic terms, the higher the ROR value, the stronger the risk of an adverse event appears to be. In absolute terms, the ROR indicates an increased risk of adverse event reporting, and not a risk of adverse event occurrence. The ROR is different from the “odds ratio” (OR) that is commonly used in epidemiological studies. Because of these deficits within the SRS, the ROR does not allow for risk quantification. Rather, the ROR offers a rough indication of the strength of the signal and relates only to the hypothesis [8]. This should be strengthened in pharmacovigilance studies.

In evaluations of the risk of LQTS, unadjusted confounding factors should be taken into account. The ROR is a clear, easy to understand, and applicable technique, which allows the control of covariates through logistic regression analysis [46–48]. However, we did not perform further analyses to obtain adjusted RORs for age, gender, and other comorbidities. The reasons were as follows: 1) the number of reports was several dozen to several hundred at most, and 2) the JADER database does not always contain enough patient background information to properly evaluate an event. Therefore, it seemed to be difficult to apply the logistic regression analysis to adjusting cofactors in this study. The covariates should be evaluated with respect to a variety of patients’ backgrounds in well-organized epidemiologic studies in the future.

Despite the inherent limitations of SRS data, the results we obtained about the association between suspected drugs and LQTS are in agreement with the results of previous reports. Our analysis of time-to-onset data indicated that the time-to-onset profiles of LQTS differed among drugs. This information may be useful for clinicians and patients to prevent sudden death following LQTS and implement more efficient therapeutic plans.

## Conclusion

Our study assessed time-to-onset data for LQTS obtained from the JADER database. The results indicated that the profiles of LQTS onset differed among drugs. Our findings corresponded with those of previous reports and confirmed the necessity of long-term observation after administration, especially for specific drugs such as bepridil and aprindine. This information may be useful for the prevention of sudden death following LQTS and for efficient therapeutic planning.
